# Comprehensive Review of Emergence and Virology of Tickborne Bourbon Virus in the United States

**DOI:** 10.3201/eid2901.212295

**Published:** 2023-01

**Authors:** Molly K. Roe, Elise R. Huffman, Yara S. Batista, George G. Papadeas, Sydney R. Kastelitz, Anna M. Restivo, Christopher C. Stobart

**Affiliations:** Butler University, Indianapolis, Indiana, USA (

**Keywords:** Bourbon virus, RNA viruses, thogotovirus, emerging virus, tickborne viruses, vector-borne infections, zoonoses, viruses, United States

## Abstract

The emergence of SARS-CoV-2 and the worldwide COVID-19 pandemic triggered considerable attention to the emergence and evolution of novel human pathogens. Bourbon virus (BRBV) was first discovered in 2014 in Bourbon County, Kansas, USA. Since its initial discovery, several cases of BRBV infection in humans have been identified in Kansas, Oklahoma, and Missouri. BRBV is classified within the *Thogotovirus* genus; these negative-strand RNA viruses appear to be transmitted by ticks, and much of their biology remains unknown. In this review, we describe the emergence, virology, geographic range and ecology, and human disease caused by BRBV and discuss potential treatments for active BRBV infections. This virus and other emerging viral pathogens remain key public health concerns and require continued surveillance and study to mitigate human exposure and disease.

## Abstract 2

This novel human pathogenic tickborne thogotovirus is found in the eastern and central regions of the country.

The first case of Bourbon virus (BRBV) was identified in June 2014 in Bourbon County, Kansas, USA, after severe febrile illness developed in a previously healthy middle-aged (>50 years of age) man (*1*). Several days after he removed an engorged tick from his shoulder, nonspecific symptoms of disease appeared. After 3 days of worsening fever, myalgia, arthralgia, and diarrhea, the patient visited his primary care physician and was prescribed doxycycline. The next day, the patient was admitted to the hospital because of dehydration, syncope, and a possible tickborne illness. Doxycycline treatment was continued; however, the patient did not respond, and symptoms continued to progress toward multiorgan failure. Laboratory results revealed progressive leukopenia and thrombocytopenia (which are now considered identifiers of potential BRBV infection). Patient blood samples tested negative for all known regional tickborne diseases. Therefore, a whole blood sample was sent to the US Centers for Disease Control and Prevention (CDC) to test for Heartland virus (HRTV), a similar emerging tickborne virus in the region. The index patient died 11 days after symptom onset.

Initial efforts at CDC to identify the causative agent of disease in this first case revealed heterologous (non-HRTV) viral plaques in plaque reduction neutralization tests performed by using serum from the deceased patient and including a control HRTV strain. Subsequent electron microscopy revealed pleiomorphic viral particles consistent with the family Orthomyxoviridae (*1*). Phylogenetic analyses revealed a close relationship between the patient’s novel virus and Thogoto and Dhori viruses, placing it within the genus *Thogotovirus.* Subsequent genetic analyses supported this initial genus classification (*2*,*3*). Recently, another novel thogotovirus (Oz virus) was discovered in ticks in Japan; this virus was capable of replicating in mammalian cell lines and is the closest known relative of BRBV (*3*). BRBV was the first human pathogen in the genus *Thogotovirus* identified in the Western Hemisphere; Aransas Bay virus, another pathogenic member of this genus, was reported in ticks found in seabird nests in the United States (*2*). Since its initial identification, >5 human cases of BRBV-associated disease have been reported in the Midwest region of the United States (*1*,*4*–*8*). Because little is known about BRBV biology and no specific treatments or vaccines are available, further studies of BRBV are needed.

## Bourbon Virus Genetics and Replication

### Genetics and Classification

**Figure 1:**
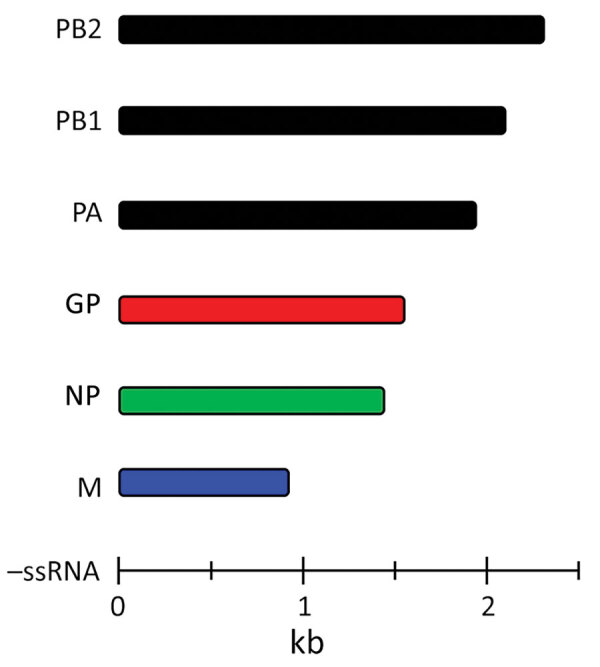
Gene segments of Bourbon virus. Bourbon virus genome comprises segmented, ≈10–11-kb, single-stranded negative-sense RNA. Specific proteins are encoded by 6 gene segments. GP, glycoprotein; M, matrix protein; NP, nucleoprotein; PA, polymerase acidic protein, PB1, polymerase basic protein 1; PB2, polymerase basic protein 2; –ssRNA, negative single-strand RNA.

BRBV consists of a segmented, ≈10–11-kb, single-stranded negative-sense RNA genome (Figure 1) (*2*,*3*). Phylogenetic analysis indicates that BRBV has the greatest similarity to Oz, Dhori, and Batken viruses within the genus *Thogotovirus* and family Orthomyxoviridae (*1*–*3*). The 6 negative-strand RNA segments of the BRBV genome encode the putative glycoprotein (GP), nucleoprotein (NP), matrix protein (M), and the 3 polymerase subunits PA, PB1, and PB2 (*2*). Gene expression and genetic organization are consistent with other orthomyxoviruses. 

The genus *Thogotovirus* contains several other emerging viruses: Araguari, Aransas Bay, Dhori (including the subtype Batken), Jos, Oz, Thogoto, and Upolu viruses (*3*). Most of these species have yet to be accepted by the International Committee on Taxonomy of Viruses. Thogoto, Dhori, and Bourbon viruses are known to cause infectious disease in humans.

Despite BRBV sharing several properties with Thogoto and Dhori viruses, such as dependence on hard ticks for transmission and similar virion structures, BRBV disease pathology remains distinct from those viruses and more closely resembles that of HRTV and severe fever with thrombocytopenia syndrome virus. However, genomic analysis of virus open reading frames (ORFs) revealed that BRBV has genetic identity with Dhori and Oz viruses ranging from ≈59% in the most divergent GP gene to ≈82% in the most conserved PB1 gene (*2*,*3*). Consequently, BRBV is classified in the *Thogotovirus* genus and recognized as a relative of both Oz and Dhori viruses.

### BRBV Virion Structure and Replication

**Figure 2:**
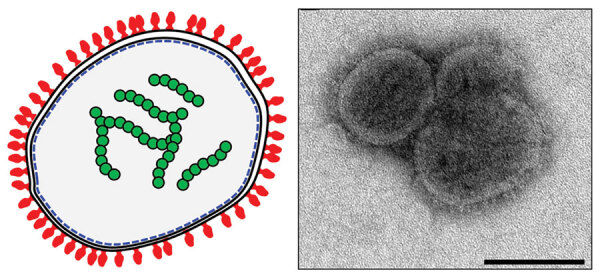
Diagram (left) and electron micrographic image (right) of Bourbon virus showing putative structural organization of the virion. Red structures represent glycoproteins attached to the outside of the virion; green structures represent the 6 RNA gene segments coated with nucleoproteins. Scale bar is 100 nm. Electron micrographic image credit: Public Health Image Library (https://phil.cdc.gov).

BRBV forms a pleomorphic (filamentous or round), ≈100–130-nm enveloped virion that is consistent with virions of other orthomyxoviruses (Figure 2) (*1*,*3*). Electron microscopy of BRBV virions shows multiple genomic segments that are likely coated internally with NPs and numerous GP molecules studding the virion surface (*1*,*5*). Although replication of BRBV has not yet been directly investigated, BRBV genetic analysis, recent crystallization of the BRBV postfusion GP, and studies of replication of related thogotoviruses provide several clues regarding the replication cycle of this virus.

All thogotoviruses use a single attachment GP to mediate virus attachment to and fusion with the host cell (*5*,*9*). The postfusion conformation of BRBV GP was recently crystallized; several structural similarities and distinct differences to previously crystallized Dhori and Thogoto virus GPs were observed (*2*,*5*). The BRBV GP is a type III fusion protein related to baculovirus Gp64 and consists of 5 distinct domains that assemble into homotrimers on the virus surface (*5*,*10*). The cell receptor for BRBV remains unknown; however, ecologic surveillance and in vitro cell culture studies collectively suggest that BRBV exhibits wide vertebrate and invertebrate species tropism (*2*,*11*,*12*). Electron microscopy and postfusion GP crystal structure suggest that BRBV attaches to host cells through a glycan-like cellular receptor and initiates entry by endocytosis (*1*,*5*).

After an endosome has formed, acidification of the endocytic compartment triggers a conformational change in the GP that causes fusion of the viral envelope with the endosomal membrane and release of the genome into the cytoplasm (*5*,*13*). All 6 genomic RNA segments are encapsidated by viral NPs, forming viral ribonucleoprotein complexes (*14*). Intracellular trafficking of viral ribonucleoprotein complexes into the nucleus, the site of replication for thogotoviruses, is driven by viral NPs, which contain a nuclear translocation signal and are known to accumulate in the nucleus during active infection (*14*,*15*). Virus replication is induced by heterotrimeric polymerase complexes formed from PB1, PB2, and PA protein subunits and is believed to be consistent with mechanisms described for other orthomyxoviruses (*16*). Similar to influenza virus and other orthomyxoviruses, thogotoviruses depend on host RNA polymerase II activity and a unique cap-snatching mechanism, whereby the viral polymerase complex cleaves the 5′-methylated cap of cellular mRNA and uses this capped leader sequence to prime viral mRNA transcription (*16*–*18*). Little is known about the nuclear export pathways of newly formed viral ribonucleoprotein complexes, but both M and NP have been implicated in aiding this process (*15*,*19*). Thogotovirus assembly and release appears to occur at the plasma membrane, activated by pH-dependent oligomerization of M particles in the cytoplasm (*1*,*19*).

## Vectors, Hosts, and Geographic Range of BRBV

### Detection and Distribution of BRBV in Invertebrates

**Figure 3:**
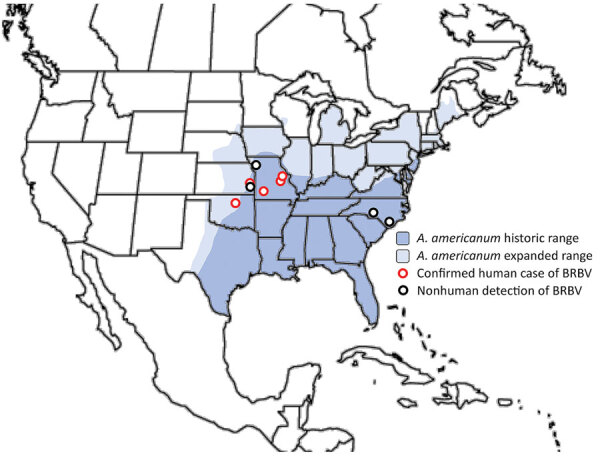
Geographic range of BRBV and its vector, the *Amblyomma americanum* tick. Confirmed human cases of BRBV infection and virus detection in nonhuman animals are superimposed over historic and expanded geographic ranges of the lone star tick (*A. americanum*). Confirmed human cases of BRBV infection were identified by the US Centers for Disease Control and Prevention, and detection of virus in nonhuman animals occurred primarily through sampling of ticks and subsequent testing by using PCR and serologic testing of mammals. BRBV, Bourbon virus.

**Figure 4:**
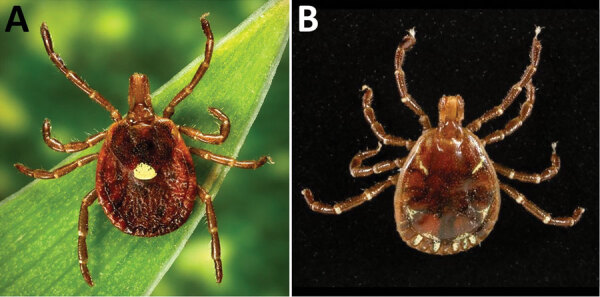
Female (A) and male (B) lone star ticks (*Amblyomma americanum*). Image credit: Public Health Image Library (https://phil.cdc.gov).

To date, all known human cases of BRBV infection have been found in 3 US states, Kansas, Oklahoma, and Missouri (Figure 3). In each case, recent tick bites were associated with the onset of disease (*1*,*8*). Since the initial identification of BRBV in a tick-infected person, several studies have used PCR-based surveillance testing to show that BRBV can be detected in all life stages (larvae, nymph, and adult) of lone star ticks (*Amblyomma americanum*) (*4*,*20*). Lone star ticks (also known as northeastern water ticks or turkey ticks) are a species of hard tick with a wide range throughout the eastern and central United States (Figures 3, 4); they are commonly found in both wooded and grassy areas and known to harbor several human pathogens, including HRTV and Tacaribe virus and the bacteria *Ehrlichia* spp., *Francisella tularensis*, *Coxiella burnetti*, and *Rickettsia amblyommii* (*21*). Although other common species of ticks have been tested at surveillance sites, such as *Amblyomma maculatum*, *Dermacentor variabilis*, *Haemaphysalis leporispalustris*, *Ixodes scapularis*, and *Ixodes dentatus*, to date, BRBV has only been found in *A. americanum* ticks (*4*,*20*). Recently, *A. americanum* ticks were shown to be capable of sustaining and transmitting BRBV through cofeeding on animal hosts (*22*). Although the *A. americanum* tick remains the only vector to harbor BRBV thus far, in vitro studies using cell culture have shown wide species tropism of BRBV. Multi-logarithmic BRBV replication was shown in the vertebrate cell lines Vero, Vero E6, LLC-MK2, BHK21Cl-15, HeLa, and HUH-7 and tick cell lines RAE/CTVM1, HAE/CTVM9, and AVL/CTVM17, indicating further surveillance will be necessary to detect additional invertebrate hosts (*2*).

### Detection of BRBV in Nonhuman Vertebrates

Similar to many other tickborne viruses, BRBV is believed to be transmitted to and amplified in nonhuman vertebrate hosts. Serologic testing was performed on a wide array of common mammal and avian fauna found near the sites of confirmed human cases in Missouri and at a distant site in North Carolina (still within the range of *A. americanum* ticks); numerous mammals were seropositive for BRBV, including domestic dogs, eastern cottontail rabbits, horses, raccoons, and white-tailed deer (*11*,*12*). The 2 most common seropositive animals identified were raccoons and white-tailed deer. No evidence of prior infections was observed in any tested bird species, suggesting that common nonhuman mammals likely serve as potential BRBV amplifier hosts (*12*).

## Bourbon Virus Disease in Humans and Potential Treatments

Five confirmed cases of BRBV infection have been reported in humans (*5*,*7*), and all 5 cases are believed to have been caused by tick bites. Although *A. americanum* ticks remain the only confirmed competent vector for BRBV, no tick species identification was made in those human cases (*22*). Limited data are available on BRBV disease in humans; however, initial symptoms of infection appear ≈2–7 days postexposure (tick bite) and include weakness, nausea, myalgia, arthralgia, fatigue, and diarrhea (*1*,*7*,*8*). Concurrently or shortly after the onset of initial symptoms, a fever and papular rash developed in all described cases. Laboratory testing of blood samples from infected persons showed consistent evidence of thrombocytopenia, leukopenia, lymphopenia, and elevated levels of aspartate transferase and alanine transferase (*1*,*8*). Late-stage BRBV disease is associated with shock, cardiac dysregulation, and pleural effusions (*1*,*7*,*8*). In confirmed fatal cases, time from initial symptoms to death was ≈11–24 days. In postmortem analysis of the index case, acute bone marrow suppression was noted (*1*).

The pathogenesis of BRBV in humans remains largely unknown. However, studies using footpad or intraperitoneal BRBV inoculations in type I interferon receptor deficient *Ifnar1*^−/−^ mice showed the virus caused active viremia and lethal systemic infection; the highest viral loads were detected in the liver and spleen, and lower viral loads were detected in the blood, kidneys, and heart (*8*,*23*). Pathogenesis observed in those mice was consistent with progressive infection from the initial entry site to multiple organs, including the liver (supported by altered aspartate transferase and alanine transferase levels), lungs (pleural effusions), and heart (cardiac dysregulation). Efforts to establish infection or lethal disease in wild-type mice were largely unsuccessful (*8*,*23*). BRBV is highly sensitive to type I and II interferons, suggesting that advanced human disease and death might be caused, in part, by existing weaknesses in antiviral innate host immunity (*23*). 

Because of the low incidence rate of BBRV infection and similarities to other tickborne diseases, additional cases of BRBV disease have likely been either misidentified or unreported. All cases to date have been confirmed at CDC by using PCR (*4*,*24*). No established treatment for BRBV disease has been reported other than supportive care. Studies in *Ifnar1*^−/−^ mice indicate that early introduction of several known antiviral treatments might be effective, including interferon-α or the viral replication inhibitors ribavirin, favipiravir, or myricetin (*8*,*23*,*25*). Recently, a reporter system for BRBV was developed, which will enable more efficient screening of putative inhibitors of this virus (*25*).

## Future Outlook

During the past 3 years, the emergence and pandemic spread of SARS-CoV-2 has highlighted the potential for evolution and proliferation of new pathogens. Thus far, BRBV has remained limited to a small number of confirmed human cases. However, many unanswered questions persist that are related to both virology and ecology of BRBV. Most of what is currently known about BRBV originates from 2 published confirmed cases of disease or ecologic surveillance studies in a small number of states in the US Midwest or North Carolina. Therefore, a substantial need remains to determine mechanisms of viral replication, detect other potential vector hosts, and conduct additional surveillance in unexplored regions within the geographic range of *A. americanum* ticks.

The *A. americanum* tick is the only known vector responsible for BRBV spread to humans. The expanding range of *A. americanum* ticks, partly driven by climate change, might lead to more exposure events (*26*). Monitoring trends in tickborne and mosquitoborne diseases has become more prominent in recent literature (*27*). Proactive safety and awareness of tickborne diseases has been encouraged, especially because ticks such as *A. americanum* continue to be dominant health threats in much of the forested regions of the United States (*21*). Because of the lack of knowledge and established treatments or vaccines for BRBV, CDC recommends using insect repellent, wearing long sleeves and pants, and conducting a thorough tick check after spending time in known tick-infested regions (*28*). 

Recent evidence suggests increasing potential for BRBV genetic evolution through recombination with related thogotoviruses. The recent discovery in Japan of Oz virus, which exhibits high sequence identity to BRBV, in *Amblyomma* sp. ticks, which share the same genus as the lone star tick, illustrates the necessity for further examination of thogotoviruses and their geographic distribution (*3*). To survive in its human host, BRBV must first overcome the interferon-induced myxovirus resistance protein A (MxA) to avoid the host’s innate antiviral defense system. Thogotoviruses are normally susceptible to inactivation by MxA, but a recent study of Jos virus showed that mutations in the viral NP can lead to resistance (*14*). Although this position is conserved among viruses in the *Thogotovirus* genus, only 1 amino acid change in the viral NP was required to fully escape MxA without replicative fitness loss. As described previously, BRBV is particularly sensitive to interferon signaling, which suggests a possible therapeutic agent for active BRBV infections (*23*). However, those studies indicate that thogotoviruses, including BRBV, might find novel mechanisms to evade host interferon-stimulated gene expression.

A substantial need exists for further research on other possible tick vectors of BRBV and the role of amplifier hosts. Seropositivity of several common animals and the ability of BRBV to replicate in multiple animal and tick cells in vitro collectively highlight the need for more BRBV surveillance to mitigate human exposure and disease. Increases in population dynamics, climate change, and vectors mean that vectorborne pathogens such as BRBV remain a major public health concern. More surveillance of both viruses and vectors will elucidate the potential for increased transmission to and pathogenicity in humans.

## Addendum

As of 2022, Bourbon virus has now been detected in the Asian longhorned tick (*Haemaphysalis longicornis*) through a surveillance study performed in Virginia, USA (*29*). This recent finding provides evidence of possible transmission of Bourbon virus in 2 separate species of ticks, *A. americanum* and *H. longicornis*. In addition, that study expands the known region of Bourbon virus to include Virginia.
